# Benzophenones in the Environment: Occurrence, Fate and Sample Preparation in the Analysis

**DOI:** 10.3390/molecules28031229

**Published:** 2023-01-27

**Authors:** Andromachi A. Gavrila, Ioannis S. Dasteridis, Alkiviadis A. Tzimas, Theodoros G. Chatzimitakos, Constantine D. Stalikas

**Affiliations:** Laboratory of Analytical Chemistry, Department of Chemistry, University of Ioannina, 45110 Ioannina, Greece

**Keywords:** benzophenones, extraction, environment, toxicity, analysis

## Abstract

The ubiquitous presence of emerging contaminants in the environment is an issue of great concern. Notably, for some of them, no established regulation exists. Benzophenones are listed as emerging contaminants, which have been identified in the environment as well as in human fluids, such as urine, placenta, and breast milk. Their accumulation and stability in the environment, combined with the revealed adverse effects on ecosystems including endocrine, reproductive, and other disorders, have triggered significant interest for research. Benzophenones should be extracted from environmental samples and determined for environmental-monitoring purposes to assess their presence and possible dangers. Numerous sample preparation methods for benzophenones in environmental matrices and industrial effluents have been proposed and their detection in more complex matrices, such as fish and sludges, has also been reported. These methods range from classical to more state-of-the-art methods, such as solid-phase extraction, dispersive SPE, LLE, SBSE, etc., and the analysis is mostly completed with liquid chromatography, using several detection modes. This review critically outlines sample preparation methods that have been proposed to date, for the extraction of benzophenones from simple and complex environmental matrices and for cleaning up sample extracts to eliminate potential interfering components that coexist therein. Moreover, it provides a brief overview of their occurrence, fate, and toxicity.

## 1. Introduction

The abundance of contaminants has already taken its toll on the environment and will continue to impact it and the ecosystems [1]. Given the severity of this issue, studies and reports are plentiful regarding their timely detection and measures for the prevention or remediation of contaminated environment [2,3]. Contaminants of emerging concern are not only understudied but also, there is no current legislation or regulation for some of them to restrict their usage [4]. Pharmaceuticals, laundry detergents, food additives, natural and synthetic hormones, pesticides, plasticizers, flame retardants, etc. [2,4], such as parabens [5], bisphenol A, 1,4-dioxane, as well as additives, and ingredients of personal care products, such as benzophenones (BPs), and many more are listed as waterborne pollutants of emerging concern [6]. They can be detected in urban, industrial, hospital, or agricultural wastewaters, entering the water cycle since they are discharged into the environmental compartments [7,8]. They are, mainly, produced by the use of commercial products or other activities and reflect the habits of modern society [6]. Even though their concentration levels in the environment are not high, their frequent occurrence and adverse impact on the environment make them important pollutants for research [9,10]. Over 700 contaminants of emerging concern, categorized into 20 classes, their metabolites, as well as their transformation products have been detected in the European marine environment [3].

The BPs constitute a group of compounds belonging to waterborne pollutants of emerging concern, with the chemical formula (C_6_H_5_)_2_CO or Ph_2_CO. They consist of two phenyl groups linked to a carbonyl group [5]. So, they are considered aromatic ketones capable of photoabsorbing in a wide range. There are various BPs, depending on the molecular substitution on the benzene rings (Table 1), which exhibit slight differences in their properties [11]. Due to their lipophilic character, they tend to accumulate in the environment, including organisms [9,11]. They also demonstrate high stability; for example, in surface waters, the half-life of oxybenzone is reported to be ~2.4 years [12,13]. BPs have been widely used because of their ultraviolet (UV)-absorbing properties, finding application as UV filters in sunscreens, cosmetics, and personal care products. Furthermore, they can be used as UV stabilizers in industrial products, such as food packaging [14,15], and additives in textiles, plastics, paints, and more [16]. They can enter the aquatic environment, mainly via direct pollution from human activities, such as swimming, especially during the summer season, and via release from wastewater treatment plants, owing to their inefficient removal [17,18]. Even though they pose a threat to the environment, there is no established regulation so far [4,5].

The reliable detection of target analytes in complex matrices depends strongly on the quality of sample preparation and separation-quantification. It goes without saying that the performance of an analytical instrumental method can be exploited fully depending on the achieved success of the applied sample preparation method [19]. In the context of sample preparation, the extraction of target BPs from a sample and the elimination of interfering components from a sample extract, the so-called cleanup, are necessary. These two steps can be either separately executed or integrated in a single sample pretreatment step. This review is meant to provide an outline of the literature with recent analytical methods for BPs putting a premium on the development and establishment of sample preparation modes and on the examination of procedural detailed to demonstrate acceptable performance.

## 2. Occurrence, Fate, and Toxicity of BPs in the Environment

In order to effectively prevent and deal with the spreading of pollutants and to be made aware of the severity of the contamination, it is important to have good knowledge of their occurrence in the environment, their fate, behavior, toxicity, and bioavailability [20]. Their occurrence in the environment depends on the way that they enter and the frequency of use. In the case of BPs, their existence is mainly due to use in consumer products and inefficient treatment of wastewater in treatment plants [3,6,13]. Their transport to the environment depends strongly, as in the case of all chemical stressors in environmental media, on their physicochemical characteristics [13]. As previously mentioned, BPs are lipophilic molecules with a wide range of octanol/water partition coefficients (Log*P_ow_*) [21] and a wide range of organic carbon partition coefficients (*K*_oc_), exhibiting medium to high solubility in water [13,22]. Structurally and in terms of their ability to accumulate, they are comparable to persistent organic pollutants [2]. There are twelve main derivatives of BPs, of which BP-1, BP-3, BP-4, BP-8, and 4-hydroxy benzophenone have been verified, in many cases [9,14,18,23]. Much research has been conducted in the aqueous environment, indicating that BPs can be detected at concentrations varying from ng/L to μg/L, hinging on the location, season, and human activity [8,23]. 

Among various BPs, much emphasis has been placed on BP-3, since it has been detected most frequently, due to its common use as a UV filter in several products for decades [24]. In 2012, it was characterized as a “high production volume chemical” [13] and recently, its use has been prohibited in Hawaii and Key West [25]. The presence of BP-3 has been reported in river water (2031 ng/L), lake water (0.2 μg/L), seawater (34.3 μg/L), groundwater (0.034 μg/L), swimming pool water (4.5 μg/L), tap water (0.45 μg/L) and wastewater influent (10.4 μg/L) [26,27,28]. Additionally, BP-3 has been detected in sediments, in Banyuls bay (49.4 ng/g) [29], Colombia (5.38 ng/g) [30], in marine sediments in California (1600 ng/g), etc. [1]. Cadena-Aizaga et al. investigated seawater from Gran Canaria Island in Spain, as well as in influent and effluent water from wastewater treatment plants (WWTPs), highlighting the presence of BP-3 [31], while in Brazil, BP-3 and BP-4 have been reported in drinking water [32]. BPs have also been found in Huangpu river in China, at concentrations up to 127 ng/L, and in farms and WWTPs, at concentrations reaching 400 ng/L. BP-3 and BP were the most frequently detected among the various BPs and their presence along with that of BP-1 and 4-OH-BP have been increasing in recent years [10]. The BP-4 has been found in river water in Switzerland [33] while it has also been detected in Spain reaching concentrations of 849 ng/L and 4858 ng/L, in rivers and wastewater, respectively [34,35,36]. BP-8 is a less-studied BP and has been prohibited as a cosmetic ingredient in Europe and Japan. Despite this, it is encountered in the environment, as related studies have shown. For example, it was found in China and Spain at concentrations of 84 ng/L and 55 ng/L, respectively [8]. In a river, in the United Kingdom close to a WWTPs, BP-4, BP-3, BP-2, and BP-1 were detected at concentrations of 0.3 mg/L, 44 µg/L, 26 µg/L, and 17 µg/L, respectively [37].

The BPs exhibit good photostability, as mentioned above [13,38]. Therefore, they are scarcely degraded upon exposure to light. For instance, the irradiation of water containing BP-3 for four weeks led to a degradation of 4% [39]. The BP-1, which is a biodegradation product of BP-3, can be photodegraded more easily, in contrast to the latter, which remains unchanged [30]. In the case of BP-8, rapid degradation in the presence of chlorine, such as in swimming pool conditions was noticed and two by-products were formed in high yield, continuing to pose a threat to the environment. On the other hand, only 50% degradation was noticed after exposure to artificial solar radiation, for 14 h [2].

As BPs are commonly used as UV filters in sunscreens, they can be absorbed into the human skin or they can be released during bathing or swimming, ending up in water [8,22]. Data concerning the American population showed that 98% of urine samples contained a detectable amount of BP-3 and its metabolites [40]. Correspondingly, research on children in Denmark revealed detectable amounts of BP-3 in the urine samples of all participants [41,42]. Its presence, also, has been confirmed in plasma [43], human brain [44], and breast milk [45,46,47], while BP-1 and BP-4 were identified in human placenta [28,48,49], and BP-4 and BP-3 in fish lipids [33] and mussels [50].

Evaluation of their toxicological impact is crucial and toxicity studies contribute to the environmental risk assessment [31]. The observed adverse effects of BPs involve disruptions in the normal functions of the endocrine system of organisms [39,51], effects on reproduction [39,52], developmental toxicity, and neurotoxicity [10,18,53]. Furthermore, the International Agency for Research on Cancer mentioned that BP is considered capable of causing human cancer as evidenced by the carcinogenicity in animals [14]. Additionally, BPs exhibit adverse effects on phytoplankton, affecting the whole trophic chain and, ultimately, the higher-trophic-level organisms [24]. A decrease in the growth of green algae *Tetraselmis* sp. was noticed and an inhibitory concentration (IC_50_) of 143 µg/L was measured after 7 days of exposure to BP-3 [54]. *Chlamydomonas reinhardtii* [55], *Photobacterium phosphoreum*, *Daphnia magna* [56], *cyanobacteria*, *Microcystis aeruginosa*, *Chlorella* sp., *Arthrospira* sp. [57] and the green alga *Chlamydomonas reinhardtii*, are also species that are affected by the presence of BP-3, at concentrations close to those found in the environment [24], while the IC_50_ values range between 100 µg/L and 20 mg/L [24,36,54,55,58,59]. Moreover, Rioboo et al. evaluated the cytotoxicity of BP-3, and BP-4 in microalga *Chlamydomonas reinhardtii*, concluding that BP-3 is more harmful to the aquatic environment. Among the reported observations are the diminished cell proliferation, due possibly to the decrease in protein F-actin, photosynthesis process alteration, decreased photosynthetic yield at concentrations of BP-4 corresponding to two toxic units, inhibition of multixenobiotic resistance mechanisms (extrusion pumps), oxidative stress, and DNA fragmentation [60].

The assessment of acute toxicity of BP-3 on *Chlorella vulgaris*, *Daphnia magna*, and zebrafish was examined revealing a high level of toxicity on the above species. The LC_50_ values for *Daphnia magna* and *Chlorella vulgaris* were 1.09 mg/L and 2.98 mg/L, respectively, and 3.89 mg/L for zebrafish. *Daphnia magna* appears to be the most sensitive of the organisms followed by *Chlorella vulgaris* and zebrafish [9]. In another study, vitellogenin 1 gene expression of zebrafish eleuthero-embryos exposed to BP-3 was affected, especially at the high concentration of 1000 μg/L, indicating estrogenic activity [61]. Male Japanese medaka and rainbow trout were affected in a similar way [62]. Eleuthero-embryos and adult male zebrafish were affected also by the presence of BP-4. The estrogenic activity and disruptions in the thyroid development of embryos, and hormonal effects on male adults were low [35]. Likewise, zebrafish exposed to concentrations of BP-3 in the range of 2.40 μg/L to 3.12 μg/L, in the early developmental stages, might present reduced hatching and deformities [10]. Decreased heartbeat of zebrafish larvae was also observed due to exposure to sediment spiked with BP-3 [1]. Furthermore, Tao et al. explored the influence of 10 μg/L of BP-3, which represents an environmentally relevant concentration in embryos of zebrafish, demonstrating a neurotoxicity effect during their development [28], while chronic exposure (¬ 5 months) of zebrafish at the same concentration of BP-3 revealed neurobehavioral effects [53]. Moreover, oxidative DNA damage and apoptosis in Chinese rare minnows due to BP, BP-1, and BP-4 exposure were reported by Yan et al. [23]. In this case, the effect of BP-1 was more intense. There are also indications that BPs exhibit in vitro estrogenic activity in the human breast cancer cell line MCF-7 [63], and exposure to BP-1 may be related to endometriosis [64]. 

## 3. Sample Preparation for Benzophenone Detection

It is easily understood that BPs constitute a class of compounds with a profound environmental impact. Therefore, their monitoring in the environment is of high importance. To this end, sensitive, rapid, and robust methods are needed, so as to obtain accurate results and introduce legislation for taking measures to avoid further environmental loading, safeguarding human health. In the following, specific features of extraction and cleanup modes and methods used in the analysis of the BPs are discussed more thoroughly.

### 3.1. Solid-Phase Extraction

Solid-phase extraction (SPE) is one of the most exploited sample preparation methods for extracting and simultaneously preconcentrating and cleaning up samples from common interfering substances of the matrix. The SPE-based procedures have been developed as the basis for the development of new analytical methods. Archana et al. developed a procedure for the detection of common pharmaceuticals, personal care products, and BPs in river water [65]. For the extraction, a portion of the sample was passed through a C18 SPE cartridge (Agela Cleanert™ ODS C18, 500 mg/5 mL) and eluted, successively, with methanol and acetone/methanol 1:1. After evaporation, the sample was injected and analyzed with a high-performance liquid chromatography (HPLC) system, coupled to a diode array detector (DAD). Recoveries were found to be between 80% and 86%. This method has the edge of short time of analysis (15 min) and the good reproducibility (relative standard deviation (RSD) of intra-day and inter-day replicate analyses was 1.69% and 2.04%, respectively). However, the linear range was rather short (2–10 mg/L) compared with other methods [65]. In another work, Chiriac et al. compared two types of SPE cartridges, in order to determine the optimum for the detection of six BPs and to develop an analytical method [66]. The authors used Strata C18 cartridges, achieving recoveries between 81.9 and 96.4%, and Strata-X cartridges (pore size of 30μm, polymeric reverse phase), attaining somewhat lower recoveries (74.0–82.6%) [66]. The developed method achieved enrichment factors (EF) of 200 and 100 for surface water and wastewater, respectively. Despite the overall good characteristics, the method was time-consuming, given that 100-200 mL of sample was passed with a flow rate of 5 mL/min (20–40 min extraction time), plus 30 min of solvent evaporation was required [66]. Kharbouche et al. proposed the use of mesoporous silica-based materials MCM-41 and MCM-41-CN(a cyanopropyl derivatized MCM-41) as SPE sorbents, for the extraction of four BPs [67]. They optimized the parameters of the method and found that the optimum pH for MCM-41 was 6.0 and for MCM-41-CN was 3.5. Salinity proved to negatively affect the extraction of 4,4’-dihydroxybenzophenone (DHBP) and BP-1 since their recoveries were found to be below 55% from river water containing 0.12% salt. With regard to the sorbent amount, it was found that even 150 mg of MCM- 41 was not adequate to recover the polar DHBP (<30% recovery). On the contrary, 50 mg of the MCM-41-CN sorbent exhibited far better performance for all BPs. Using the MCM-41-CN as a sorbent, from 100 mL of sample spiked with 0.1 ng/mL of each BP, 74.8–106.4% recoveries were achieved along with an EF of 100. Quantification with the standard addition method revealed a matrix effect (ME) of 3.1–14.8% for the examined BPs. The intra-day precisions of the method were between 6.0 and 15.5 (spiked with 0.1 ng/L BPs) and from 8.2 to 10.8 (spiked with 0.5 ng/L). This hints towards a rather irreproducible method [67]. Likewise, Sun et al. proposed an extraction method using molecularly imprinted polymers as SPE sorbents, for the analysis of tap water and river water [68]. As template they used BP-2, as functional monomer 4-vinylpridine, as a cross-linking monomer ethylene dimethacrylate, and as initiator they used 2,2-azobisisobutyronitrile. The sorbent material was first compared with the non-imprinted analogue and the results proved worse. Elution of the BPs from the sorbent was carried out using methanol/trifluoroacetic acid while after evaporation, the compounds were dissolved in a methanol/water mixture and injected into an HPLC-DAD system. It is noteworthy that the method was not found to be impacted by the sample pH (within the range of 3.0–9.0), thus avoiding a relevant step commonly used in many extraction procedures [68].

In another study, Narloch and Wejnerowska compared the classical SPE procedure with a microextraction with packed sorbent (MEPS) procedure [16]. The SPE method was found to have the edge over MEPS in terms of sensitivity and EF. Nonetheless, the MEPS method was much easier, faster (only 10 min was needed) and the material could be reused up to 100 times, thus lowering the total cost significantly. Moreover, MEPS used a small volume of sample, which can be an advantage when the sample is sparse, or a small volume of elution solvent is needed, thus making the method greener and reducing the overall cost. However, the drawback of low EF cannot be overcome by increasing the sample volume. The SPE method achieved an intra-day precision of 7.7% to 11.8% and inter-day precision of 8.0% to 13.4%. Likewise, the MEPS method achieved an intra-day precision of 6.6% to 15.6% and inter-day precision of 6.6% to 18.8%. Therefore, both methods exhibited mediocre repeatability and reproducibility [16].

In another study, an automated procedure of on-line SPE and LC-MS was proposed for the detection of eight BPs [69]. Different sorbents for the SPE were examined: Oasis HLB (an N-vinylpyrrolidone and divinylbenzene macroporous co-polymer), HySphere Resin GP (polydivinyl-benzene), Hysphere end capped octadecyl C18, and cross-linked styrene/divinylbenzene (PS/DVB), with PS/DVB being the best in terms of LODs, peak shapes, and recoveries. Moreover, negligible ME was recorded for groundwater and river water. Out of the five groundwater samples four contained BP3, BP1, DHBP, BP4, and BP3, while BP1 and BP4 were also detected in the river water samples. One of the main advantages of this separation and analysis method is that it offers automation, requires only a filtration step, and spiking with internal standards. The turnaround time of the on-line SPE method was 20 min. Recoveries of 88–114% for groundwater and 82–111% for river water were achieved. The developed on-line SPE method also had the advantage of an extraction time equaling analysis time, therefore making it possible for automation of the process and analyzing samples sequentially [69].

When the matrices to be analyzed are more complex, another cleanup step needs to be introduced to eliminate potential interferences. In their work, Wang and Kannan extracted BPs from wastewater and sludge from WWTPs and determined them [70]. Firstly, aliquots of water samples were separated from the suspended particulate matter, and then they were loaded on SPE cartridges, eluted with methanol and concentrated to a final volume using a nitrogen stream. For the sludge sample, an additional step of solid–liquid extraction was needed before applying SPE. Briefly, after freeze-drying of samples, the analytes were extracted with methanol/water (5:3) and after centrifugation and concentration, they were acidified with 0.2% formic acid prior to undergoing the above SPE procedure. The suspended particulate matter followed the same procedure as in sludge samples. The two extractions were enough to sufficiently extract the BPs from the sludge sample. The absolute recoveries of BPs were found in the ranges of 84–105%, 99–108%, and 81–122% for sludge, suspended particulate matter, and wastewater, respectively. It is noteworthy that this is the only work reporting the analysis of suspended particulate matter from the samples, at particularly low LOQs (0.25–0.50 ng/g) [70]. In relation to more complex matrices, Han et al. used animal and vegetation seafood as substrate for BPs detection using pressurized liquid extraction, a fast and green method for extraction, followed by a cleanup step with a mixed-mode cationic exchange SPE step for the removal of co-extracted compounds [71]. A mixed-mode cationic exchange was chosen over lipophilic balance and C-18 SPE, as recoveries for all BPs were superior. Additionally, after a comparison of the pressurized liquid extraction with Soxhlet and ultrasound-assisted methods, the recoveries were proved to be better with the pressurized liquid extraction (90.6–107.8%) compared with 83.5–88.7% and 81.4–85.2% for the other two methods, respectively [71]. Another SPE method using Oasis cartridges packed with 100 mg HLB sorbent was proposed by Luki et al. to preconcentrate and detect four BPs [72]. The working parameters were optimized by an experimental design which revealed that the elution solvent and percentage of methanol and pH were the influencing factors. In their work, Cadena-Aizaga et al. used an SPE method to extract eight organic UV filters, including BP-3, from seawater and wastewater samples [31]. The pH was adjusted to acidic values with formic acid and C18 cartridges were used, in the absence of salt. The best eluent and pH were MeOH:ACN (1:1, *v/v*), pH = 3 for seawater and MeOH, and pH = 7 for the wastewater, thus achieving preconcentration factors of 140 and 50, respectively. Table 2 summarizes the features of relevant sample pretreatment methods based on SPE.

### 3.2. Dispersive (Magnetic) Solid-Phase Extraction

Another commonly used method of extraction is the dispersive solid-phase extraction (DSPE). The materials used were specifically synthesized with high surface becoming more available to sorb the target analytes and were dispersed in the sample, providing ease of separation by centrifugation. In this context, Qiu and Ding synthesized zeolitic imidazolate framework-8 (ZIF-8) to extract BPs from surface, river, and seawater samples [73]. The ZIF-8 was prepared simply by grinding zinc oxide and 2-methylimidazole and then, mixing it with a small amount of deionized water. After optimization of the DSPE procedure, the synthesized material was added to the water samples, ultrasonicated, and centrifuged. To remove the BPs from the ZIF-8, methanol was used. The recoveries of BPs from real samples spiked with the BPs were found between 81.2 and 94.1% [73]. In another work, Wang et al. synthesized nanocomposite microspheres from polyaniline and core–shell silica mesoporous (CSMS) to extract BPs from environmental water samples [74]. They first synthesized the CSMS microspheres, which were then used to create the CSMS@polyaniline nanocomposite microspheres. River, swimming pool, and snow water samples and domestic sewage were filtered before the extraction by DSPE. The pH of the samples was optimized at the value 7.0 with NaOH and HCl, and then, the pretreated with acetonitrile and water microspheres were added. The analytes were eluted using methanol, evaporated, and redissolved in methanol. Finally, analysis was carried out on a sheathless capillary electrophoresis (CE)-MS/MS apparatus. One of the interesting findings showed that extraction efficiency lowered as the salt concentration increased. Although polyaniline can be used to extract BPs, the modified CSMS@polyaniline multiplied the recoveries of BPs by 2.0 to 3.8 times. The EF values were found to be between 470 and 660, and recoveries in the range of 84.2–101.0% [74].

The sorbent used for a DPSE procedure can be a magnetic material, which simplifies its isolation from the solution just with the use of an external magnet. This procedure is also known as magnetic DSPE (mDSPE). Making use of this procedure, Piovesana et al. proposed a magnetic graphitized carbon black adsorbent, stable in water, to be used in an mDSPE [75]. To address the common problem of background contamination in trace detection of UV filters in the environment, solvent blanks were analyzed for every batch. Furthermore, procedural blanks and two spiked samples of the highest and lowest concentration of the calibration plot were analyzed. The authors claimed that the method was fast but the analysis time took more than 60 min, while no method optimization was carried out. So, a better overview of the parameters that affect the method is lacking [75]. Similarly, Li, et al. modified commercially available Fe_3_O_4_ magnetic nanoparticles to extract BPs from soil samples [76]. Each sample was finely ground, and mixed with methanol and after centrifugation, the solvent was retracted and evaporated. After the addition of methanol, a small aliquot of the sample was diluted with H_2_O. For the extraction, 5.0 mg of the synthesized MOF-1210(Zr/Cu)-Fe_3_O_4_ was added to 20 mL of the sample containing the BPs and after pH adjustment to 6.0, NaCl solution (1%) was added. After extraction and elution with 2% formic acid–acetonitrile (v/v), analysis was carried out on an HPLC-UV system. The method achieved recoveries between 87.6 and 113.8%, and EF was found to be in the range of 91–122 [76]. In their study, Medina et al. synthesized magnetic graphene oxide composite (Fe_3_O_4_@SiO_2_@(3-aminopropyl)triethoxysilane@GO) and used it for mDPSE. During the optimization of the method, it was found that salinity could aid the extraction by salting out the compounds or hinder it by increasing the viscosity of the solution. Thus, 4% NaCl w/v was the optimum salt concentration. An acidic pH value was selected as optimal, as the surface of graphene oxide and BPs are neutral, thus promoting the hydrophobic interactions between them. On the other hand, extremely alkaline pH would damage the sorbent. Moreover, high sample volumes (i.e., 100 mL), 20 mg of sorbent, and combined vortexing and ultrasound agitation (5 min each) were found suitable to increase the extraction. The low extraction time (¬ 15–20 min) is a great alternative to the tedious SPE procedures. The material could be reused up to four times without the loss of sensitivity [77]. The downside of this method in comparison to the others mentioned is the high LOD (2500–8200 ng/L).

Our group synthesized a magnetic Fe-Cu bimetallic nanomaterial to remove different hazardous organic micropollutants, including BP-2 and BP-6 from effluent from a WWTP [78]. The nanomaterial was made by mixing (NH_4_)_2_Fe(SO_4_)_2_·6H_2_O and CuSO_4_·5H_2_O with a NaBH_4_ solution. The final product was removed from the solution washed with DDW and ethanol and dried. The samples from the WWTP were filtered, and then, the nanomaterial was added. After stirring, the latter was removed with the use of a magnet and the supernatant was analyzed to evaluate the removal efficiency. To elute the absorbed compounds from the nanomaterial, acetone containing 5% *v*/*v* formic acid was used and the eluent was also analyzed with HPLC. Different tests showed that the preferred extraction pH is slightly acidic but a change to a slightly alkaline environment caused insignificant variation on efficiency. As far as the salt concentration is concerned, the highest removal efficiency of 81% was found when 10% *w*/*v* Na_2_SO_4_ was present [78]. Table 3 summarizes the features of relevant sample pretreatment methods based on DSPE.

### 3.3. Liquid–Liquid Extraction

Despite being a classical extraction technique, liquid–liquid extraction (LLE) procedures are still being developed for the extraction of BPs from various samples. Zhang et al. proposed the use of an LLE extraction, coupled with an SPE cleanup step and HPLC-MS/MS [79]. Using 6 mL of methanol: ethyl acetate (15:85 *v/v*), recoveries greater than 80% from sediments and >70% from sludge, for all BPs tested (except BP-3), could be achieved. Neither solvent volume nor methanol enhanced the recovery of BP-3 (<44%). It was speculated that either incomplete extraction from the matrix or transformation during the cleanup step was the main reason [79]. Only BP-3 was detected in the six Songhua River sediment samples (mean concentration: 0.38 ng/g of dry weight) and all BPs were detected in Saginaw and Detroit Rivers. Sludge from Northeastern China contained BP-3, BP-1, and 4-OH-BP.

Wang et al. proposed a dispersive liquid–liquid microextraction (DLLME) procedure, using a hydrophobic deep eutectic solvent (DES) that was formed in situ, when a hydrogen bond donor and acceptor were present [80]. BPs were extracted in the droplets of the DES, which could easily be collected after solidification/floating at 22 °C. This method is one of the most facile, fast, and green, as it consumes little to no organic solvent. Intra-day and inter-day precision values were 2.0–6.1% and 3.9–7.7%, respectively, making it one of the methods with the best repeatability and reproducibility. The extraction efficiency was not affected by any changes in the pH of the sample within the pH range of 2–10, avoiding the need for pH adjustment. Moreover, salinity was not found to affect the extraction procedure, thus, making it possible for application to samples with high salinity [80].

Another DES-based method was implemented by Wang et al. for an ultrasound-assisted DLLME [81]. The inter-day and intra-day RSD values were less than 5.9%, rendering it one of the most repeatable and reproducible methodologies, with EF values between 67 and 76. The optimum amount of DES used was 30 mg since greater partitioning of the BPs in the DES was recorded. By assisting the extraction procedure with ultrasounds, the high viscosity of the DES was not a problem, resulting in a 5 min extraction procedure. Concerning salt, it was found that the addition of 1% NaCl not only aided the extraction but also promoted phase separation. Increased salt concentrations (above 3%) resulted in increased viscosity of the solution, inhibiting the extraction by decreasing the diffusion rate and partitioning of BPs. By analyzing spiked river water samples, it was found that the ME was negligible in the proposed method. The DES employed in this study was able to replace the costly and more difficultly synthesized imidazolium-based ionic liquid, commonly used in such procedures [81]. In a similar work, Çabuk and Kavaracı used an LLME method to extract BP-1, 4-OH-BP, and BP-3 from tap water, stream water, and seawater [82]. They used di-(2-ethylhexyl)phosphoric acid (DEHPA), which can change its hydrophilicity depending on the pH of the solution. By adding a small amount of DEHPA to standard solutions or samples, microdroplets were formed under certain conditions that entrapped the BPs. Adding a small amount of Fe_3_O_4_ ¬NPs made it possible to extract the BPs, which were eluted with acetonitrile. The EF values were 18–25 and the extraction recoveries were between 69 and 93% [82]. Another optimized DLLME method that uses an ionic liquid, in situ-formed, based on didecyldimethylammonium chloride (DDAC) was proposed by Ziemblińska-Bernart, et al. They concluded that the optimal ratio of DDAC to NaClO_4_ was 1:2. Samples taken from a depth of 15–30 cm from lakes and recreational beaches were free of the examined BPs [83]. The in situ formation coupled with the magnetic retrieval makes this method appealing but as mentioned above, DLLME methods using IL can be substituted for using DES. This method needed only 5 min to be completed and, at the same time, achieved the lowest LLE reported LODs.

Finally, using solidified droplets as the extracting phase, Zhang et al. used α−terpineol, a naturally occurring monoterpene enol, to extract 4 BPs from water samples. The EFs were between 29 and 47 and the recoveries ranged from 80.2% to 108.4%, with RSDs (intra- and inter-assay) less than 8.5%. [84]. The employment of α−terpineol provided a simple and rapid alternative for the determination of benzophenone compounds in aqueous samples. Table 4 summarizes the features of relevant sample pretreatment methods based on LLE.

### 3.4. Other Methods

Other methods have also been developed to extract and analyze BPs. Xu et al. synthesized a Zn-Tb heterometallic coordination polymer in order to detect BP [85]. The coordination {[Tb_2_Zn(L)_4_(H_2_O)_8_]·8H_2_O}_n_ polymer showed four emission peaks at 491, 546, 585, and 622 nm when excited at 270 nm. A quenching effect appeared when BP was present while other interfering substances showed little or no effect on the intensity of the fluorescence. The higher the concentration of the BP used, the higher the quenching effect observed. It is worth mentioning that the coordination polymer could be recovered and reused for up to six cycles just by washing it with water [85]. Although the method does not exhibit as good LODs as other methods (329 ng/L), it shows that the fluorometric detection of BPs is also possible. 

An extraction and simultaneous cleanup step assisted by ultrasound was proposed by Sánchez-Brunete et al. [86]. In this procedure, a glass column containing two circular 2 cm diameter filter papers was packed with anhydrous sodium sulphate and C18. Then, an amount of sieved sediment or soil sample was placed inside the column. The whole system was placed inside a sonication water bath supported by a tube rack. Through the formed packed column, a mixture of ethyl acetate:methanol (90:10, *v/v*) was passed, while sonicating, in order to extract the BPs and isolate them from the matrix. Although ethyl acetate eluted most of the BPs, the most polar ones such as BP-8 and BP-6 were better eluted by a 90:10, *v/v* ethyl acetate:methanol mixture. Matrix effects of up to 27% were nullified by the use of internal standard. The authors investigated the effect of moisture and found that it had no effect on the recoveries. Similarly, a residence time of 72 h between spiking and analysis had no effect on the recoveries. Furthermore, the RSD values of 3.4% to 7.5% for the intra-day precision indicated good repeatability [86]. Finally, as this method utilizes a GC-MS system for analysis, the low thermal stability and volatility of the BPs necessitate a derivatization step, thus complicating the procedure [86]. In another study, Camino–Sánchez et al., proposed an ultrasound-assisted extraction of five BPs from soils and wastewater-treatment-plant compost, with methanol as a solvent for extraction [87]. The focus of the study was the leaching of BPs in soil and compost-amended soil. The studied plots of soil were tested for 12 consecutive days and at different depths, at 10 cm intervals, reaching 60 cm. All BPs except BP-3 did not reach 60 cm, indicating good behavior, as its disposal cannot contaminate the aquifers. In compost-amended soils, leaching was almost the same as in the non-amended ones. The method’s intra-day precision was poor, up to 17.2% for soil samples and up to 20.7% for compost samples. The inter-day precision values were also high, up to 11.4% for soil and up to 13.3% for compost. This method did not use any further extraction procedure to free the sample from interferences or preconcentrate it. Matrix-matched curves were used to overcome the matrix effects.

Modified stir bars can also be used for BP absorption. Merib et al. created a cork-powder-coated polypropylene fiber material for stir bars, which was used for bar adsorptive microextraction (BAmΕ) of BP from aqueous samples [88]. The authors reported the optimal extraction time for BP to be 90 min. They opted for a 120 min extraction time as a middle ground for BP, triclocarban, and paraben extraction. For the purpose of this review, the 90 min extraction time for BP is further discussed. In this framework, not only the extraction step is time-consuming but also the 30 min desorption time adds up to the already time-consuming procedure. In a positive light, the BAmE procedure needs low extracting solvent when compared to traditional SPE, eliminating the need for laborious evaporation under a nitrogen stream. It is noteworthy that as the bar operated well below its saturation point, the authors found that by decreasing the bar length to half and by increasing the desorption volume by 2.5 times, they could improve the LOD by 2.5 times [88]. Similarly, Almeida et al. compared polymers and activated carbon coating for the BAmΕ of BPs [89]. P2 (a modified pyrrolidone polymer with a surface area of 800 m^2^/g, pore size of 85 Å, and 33 μm particle size) and AC4 (an activated carbon with a surface area of 1400 m^2^/g) polymers were compared with each other as being the two best representatives. The P2 polymer was chosen over AC4 for sampling since both the extraction (4 h as opposed to 16 h) and back extraction (15 min as opposed to 30 min) were faster. Furthermore, it provided better recoveries. The P2-coated bars were stable at pH 2–14, but the range 2–5.5 was chosen in which BPs are neutral [89]. In their work, Liu et al. proposed another stir-bar sorptive extraction (SBSE) method in soil [90]. Soil samples needed to be separated from other materials, dried to constant weight, and then pulverized and sieved, before being extracted with methanol with sonication, followed by centrifugation. This procedure was repeated twice, and the extracts were added and evaporated with a rotary evaporator and redissolved in methanol. This procedure takes about 90 min to be completed. Taking into consideration the 2-h step of SBSE and the requirement for analyte desorption, the overall procedure takes almost 4 h, thus making it an unequivocally lengthy method. The sample volume was adjusted to 15 mL, as it provided a great balance between EF values, sample consumption, and extraction time. The authors investigated the addition of acetonitrile in the sample and found that it promoted BP dispersion and avoided adsorption on the container, while it increased their solubility (optimal 0.2% *v*/*v*). They also found that 2% NaCl benefited the process and they achieved EF of 49–102 [90].

In the study of Celeiro et al., three coated sol–gel fabric phases, i.e., nonpolar, medium-polar, and polar, were fabricated and compared [91]. The highest extraction of BPs was achieved with the non-polar poly-dimethylsiloxane coating. A major drawback of poly-dimethylsiloxane coating in regular SPE is that it is highly viscous, hindering the analyte migration and increasing the sorption time. The sol–gel coating resulted in a thin film that was finely integrated into a silica fabric phase. The substrate aided the extraction by bringing the analytes close to the sorbent to interact with various interactions. With the proposed sorbent material, only 20 min are needed for the extraction. On top of that, high reproducibility of the synthesis and analysis were recorded. Although this study detected UV filters in real samples, BP-3 was not one of them [91]. 

Huang et al. in their novel work, developed a Quick, Easy, Cheap, Effective, Rugged, and Safe (QuEChERS) method to extract ten different BPs from fish samples and compared the developed method with an SPE one [14]. For the QuECheRS method, the use of ACN with 1% acetic acid, anhydrous MgSO_4_, NaCl, Sepra C18-e (50 μm, 65 Å), and Superclean primary secondary amine was proposed after optimization. At first, they tested the use of DSPE-enhanced matrix removal for the fish samples with lipid content higher than 5% but it seemed to decrease the recovery of the BPs. They also tested an SPE extraction method with PRiME HLB and SPE-C18 cartridges, but the extraction results were lower than those with QuECheRS method, with recoveries between 55% and 154%. The repeatability and reproducibility of the method were remarkably low, with RSDs reaching 26.6% and 29.3%, respectively, for the detection of the BPs [14]. This method showed that the detection and extraction of BPs are possible even with complex matrices, such as fish but further optimization is needed to increase its repeatability and reproducibility. Table 5 tabulates the most important features of the above sample pretreatment methods.

## 4. Conclusions and Outlook

A remarkable increase in the number of publications employing novel pretreatment techniques for the determination of BPs, in several matrixes, has been observed in recent years. These methods are quickly replacing conventional treatment processes developed over the last few years (such as mechanical shaking, stirring, Soxhlet, etc.), and hence, new ones have been adopted for solid, semi-solid, and water samples. A wide range of environmentally friendly solvents have been employed (including ionic liquids, deep eutectic solvents, etc.) and an even wider range of materials have been examined including polymers, carbon-based nanomaterials, etc. The combination of the above with well-established sample preparation procedures (such as, SPE, DLLME, etc.) and chromatographic techniques have contributed to the progress of this research field. For future studies, the large-scale synthesis of the examined sorbent materials should also be examined for routine analysis.

Given the widespread use of BPs, it is expected that their concentrations in the environment will be increasing in the coming years. Depending on the characteristics of the target samples, such as the nature and availability of the sample matrix, routine monitoring test methods should consider the requirements for analytical sensitivity, accuracy, and capability for sample throughput, robustness. In this context, the SPE is a straightforward and fast sample preparation method for BPs with applications to various sample matrices. It holds the largest share of the sample pretreatment methods for BPs and it is considered to be the best choice in establishing an analytical method. However, applications to analytically challenging samples that struggle to remove the sample matrix can make the well-established the QuEChERS a core component of sample preparation.

Studies about the fate and adverse impact on human health and aquatic life are rather limited. For risk assessment, challenges revolve around the extrapolation of their effects observed in individual organisms or species in the laboratory to field studies, where multiple stressors occur. While it is informative to know the effect of a single chemical parameter, its contribution in a mixture of a multitude of chemicals will be significantly influenced in the presence of them. Therefore, the monitoring of pure samples of BPs may have to be accompanied by a determined, possible, or suspected mixture of chemicals to paint the real-life scenario. In this framework, effect-based monitoring should be carried out for the assessment of water quality status and determination of the efficiency of water treatment as well as for the quantification of contaminants in aquatic environments. Despite the relatively slow progress in the field, it is expected that this topic will rapidly develop, given the harmful impact of BPs on the environment and the need for improved analytical tools.

## Figures and Tables

**Table 1:** Molecular formulas, structures, and octanol/water partition coefficients of BPs (https://pubchem.ncbi.nlm.nih.gov/ accessed on 24 November 2022).

BPs	Molecular Formula	Structure	Log*P_ow_*	CAS Number
Benzophenone	C_13_H_10_O	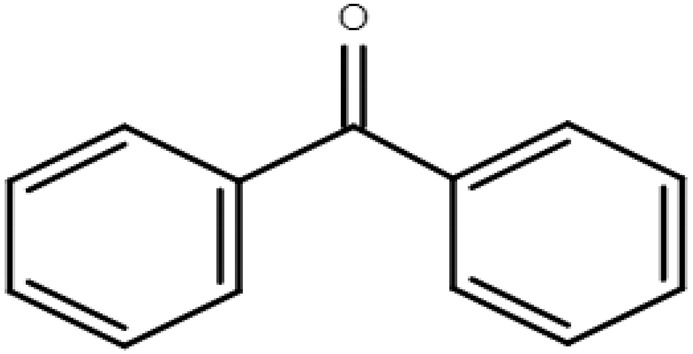	3.18	119-61-9
2,4-dihydroxybenzophenone or Benzophenone-1(BP-1)	C_13_H_10_O_3_	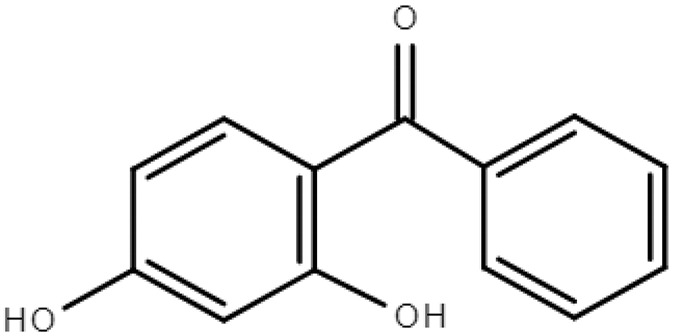	2.96	131-56-6
2,2’,4,4’-tetrahydroxybenzophenone or Benzophenone-2 (BP-2)	C_13_H_10_O_5_	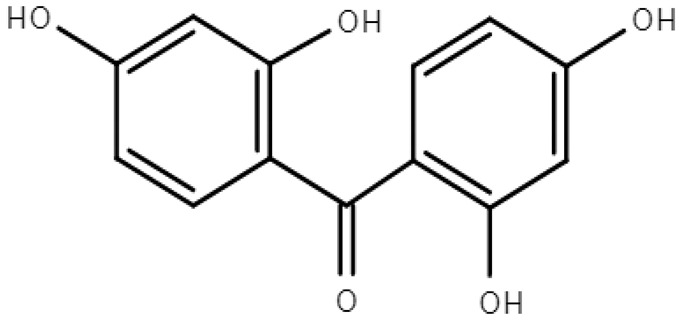	2.78	131-55-5
2 -hydroxy-4-methoxybenzophenone or Oxybenzone or benzophenone-3 (BP-3)	C_14_H_12_O_3_	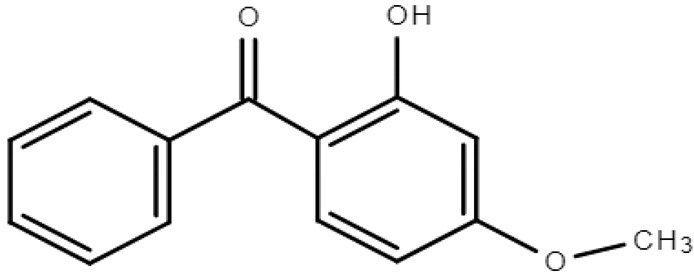	3.79	131-57-7
2-hydroxy-4-methoxybenzophenone-5-sulfonic acid or sulisobenzone or Benzophenone-4 (BP-4)	C_14_H_12_O_6_S	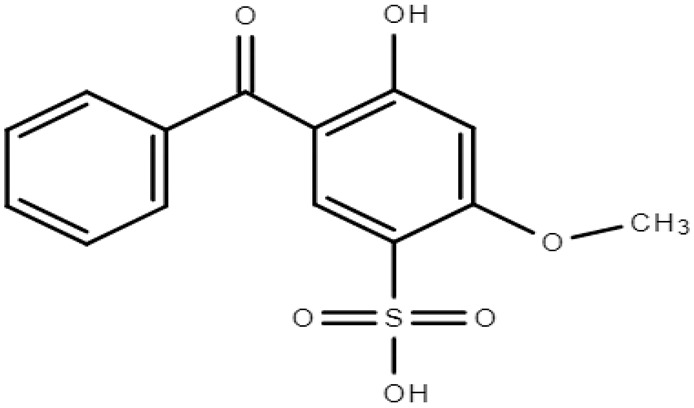	0.37	4065-45-6
2-hydroxy-4-methoxybenzophenone-5-sodium sulfonate or Benzophenone-5 (BP-5)	C_14_H_11_NaO_6_S	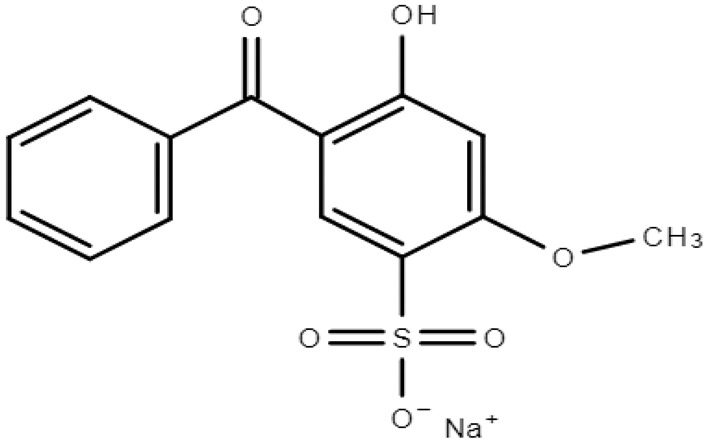	-	6628-37-1
2,2’-dihydroxy-4,4’-dimethoxybenzophenone or Benzophenone-6 (BP-6)	C_15_H_14_O_5_	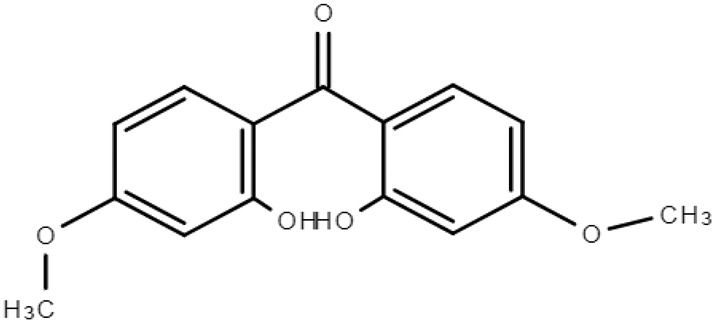	3.90	131-54-4
5-chloro-2-hydroxybenzophenone or Benzophenone-7 (BP-7)	C_13_H_9_ClO_2_	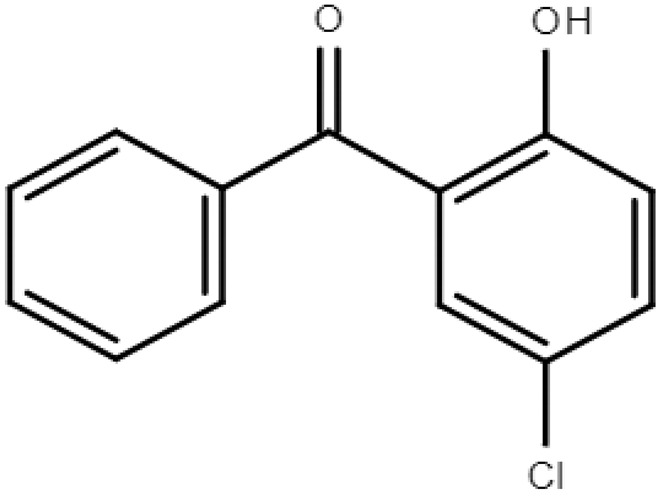	4.09	85-19-8
2,2’-dihydroxy-4-methoxybenzophenone or dioxybenzone or Benzophenone-8 (BP-8)	C_14_H_12_O_4_	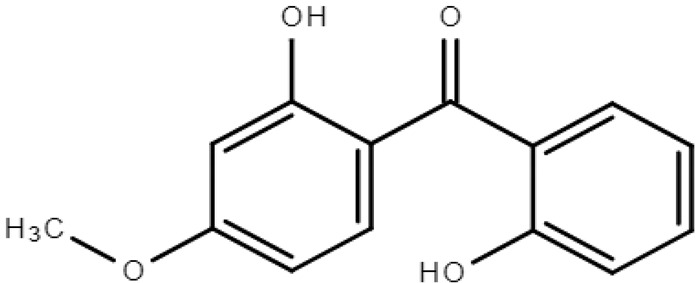	3.82	131-53-3
2,2’-dihydroxy-4,4’-dimethoxy benzophenone-5,5’-disodium sulfonate or Benzophenone-9 (BP-9)	C_15_H_12_Na_2_O_11_S_2_	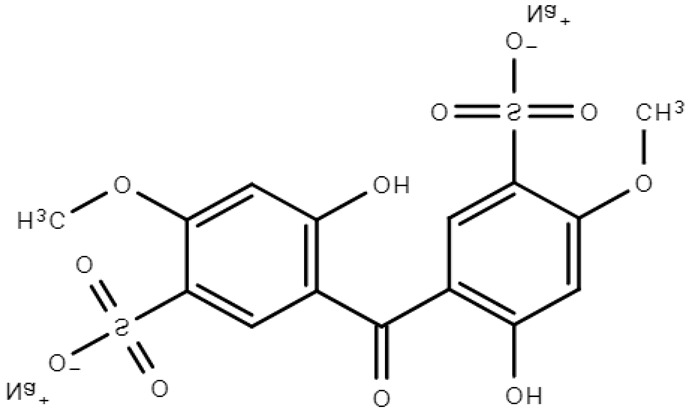	-	76656-36-5
2-hydroxy-4-methoxy-4’-methylbenzophenone or Benzophenone-10 (BP-10)	C_15_H_14_O_3_	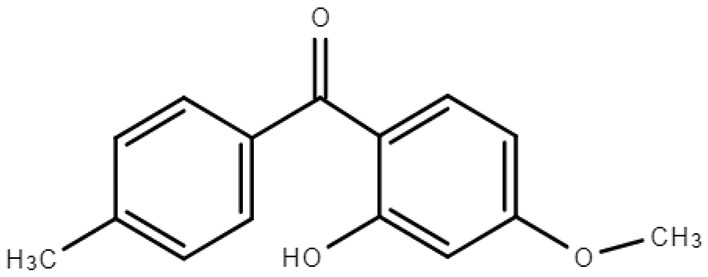	4.07	1641-17-4
2-hydroxy-4-octoxybenzophenone or benzophenone-12 (BP-12)	C_21_H_26_O_3_	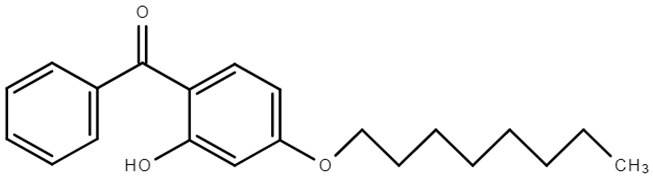	6.96	1843-05-6
4-hydroxybenzophenone	C_13_H_10_O_2_	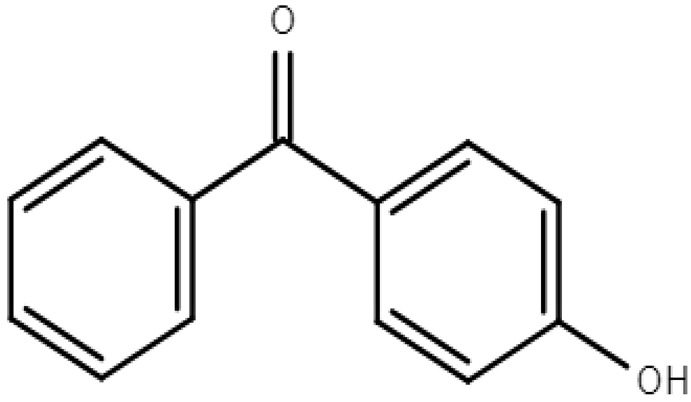	3.07	1137-42-4
4,4’-dihydroxy-benzophenone	C_13_H_10_O_3_	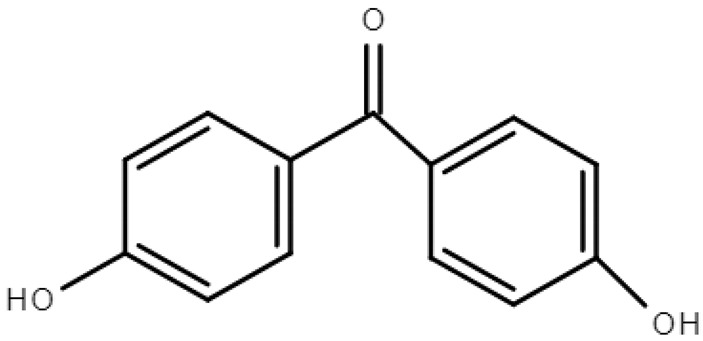	2.19	611-99-4

**Table 2:** Sample preparation methods based on SPE procedures for BP detection.

Method	Sample	Sorbent	Analytical Technique	BPs	Time for Treatment)	LOD (ng/L)	Recoveries % (RSD%)	Reference
SPE and MEPS	Groundwater, river	SPE(C-18) MEPS (syringe packed with C-18)	GC-MS	BP-1, BP-3, BP-8	-	34–67	96–107	[16]
					10	1800–3200		
SPE	River	MCM-41/ MCM-41-CN	UHPLC–MS	BP-1, DHBP, 4-OH-BP	~20 (3 days for synthesis)		74.8–106.4	[67]
On-line SPE	River Groundwater, effluent	cross-linked styrene/divinylbenzene polymer	LC-MS/MS	BP-3, BP-1, 4-OH-BP, DHBP, BP-8, BP-2BP-4	20	0.3–4	70–114	[69]
SPE	Surface water wastewater	C18	LC-MS/MS	2-OH-BP, 4-OH-BP, BP-2, BP-1, DHBP, BP-8	70	0.59–1.46 1.17–2.93	79-98	[66]
					50			
SPE Solid–liquid extraction	Sludge	methanol	HPLC-MS/MS	BP-3, 4-OH – BP, BP-1, BP-2, BP-8	~ 210	-	84–105	[70]
	suspended particulate matter				>210		99–108	
SPE	wastewater				~145		81–122	
SPE	River water	C18	RS-HPLC-DAD	BP	~120	1480	80–86	[65]
Pressurized liquid extraction and SPE	Seafood	C18	LC-QqLIT-MS/MS	BP-1, BP-2, BP-3	-	-	80.6–107.8	[71]
SPE	River water Tap water	Molecularly imprinted polymer	HPLC-DAD	BP-2, BP-1, BP-8, BP-6	~50 h (for synthesis) ~40 mins (for extraction)	250–720	86.9–103.3	[68]
SPE	Lake water	Oasis HLB 6 mL Vac Cartridges (100 mg sorbent)	LC-MS/MS	BP-1 BP-3 BP-4 4-OH-BP	280	0.04–4.4	62–82	[72]
SPE	Seawater	C18	UHPLC MS/MS	BP-3	-	11.3–36.4	43.3–100	[31]
	Wastewater					24.6–555.6	26.0–98.5	

LC-QqLIT-MS/MS: liquid chromatography–quadrupole linear ion trap–tandem mass spectrometry.

**Table 3:** Sample preparation methods based on DSPE and mDSPE procedures for BP detection.

Method	Sample	Sorbent	Analytical Technique	BPs	Time for Treatment (min)	LOD (ng/L)	Recoveries %	Reference
Magnetic DSPE	Lake water	Magnetic (Fe_3_O_4_)-graphitized carbon black (mGCB)	UHPLC-(QqQ) MS-MS	DHBP, BP4, BP-2, 4OH-BP, BP-1, BP-8, BP-3	~120 (2 days for synthesis)	1–5	85–114	[75]
Magnetic DμSPE	Swimming pool water	(Fe_3_O_4_@SiO_2_@APTES@GO)	HPLC–(QqQ)MS/MS	4-OH-BP, BP-8, BP-3, BP-6, BP-1	~15 (4 days synthesis)	2500–8200	86–105	[77]
DSPE	Surface water River water Seawater	ZIF-8 + methanol	UHPLC-QTOF-MS	BP -3, BP-8, 2-OH-BP, 3-OH- BP, 4-OH-BP	~12 (~75 for synthesis)	0.1–7	81.2–94.1	[73]
MSPE	Soil	MOF-1210 (Zr/Cu)-Fe_3_O_4_ + 2% formic acid-acetonitrile	HPLC UV	BP-1, BP-3, BP-6	~62 (>4 days for synthesis)	10–20	87.6–113.8	[76]
DSPE Fixed Bed	Effluent from WWTP	Acetone—5%, formic acid, Fe-Cu nano	HPLC-DAD	BP-2, BP-6	~ 20 (~24 h for synthesis)	-	84–92	[78]
DPSE	River, swimming pool snow water domestic sewage	CSMS@ polyaniline + Methanol	CE-MS/MS	BP-1, BP-2, BP-3, BP-6, BP-8, DHBP	~30 (>4 days for synthesis)	0.6–200	84.2–101.0	[74]

**Table 4:** Sample preparation methods based on LLE procedures for BP detection.

Method	Sample	Sorbent	Analytical Technique	BPs	Time for Treatment (min)	LOD (ng/L)	Recoveries %	Reference
DLLME	Water samples	Hydrophobic DES	HPLC-DAD	BP-1, BP-2, BP-3, BP-6	~10–15	600–1500	73.1 to 99.8	[80]
DES-ultrasound-assisted DLLME	River water	DES	HPLC-UV	BP-1, BP, BP-3	~10–15	150–300	90.2–103.5	[81]
LLE and SPE	Sediment	Methanol (LLE) oasis HLB (SPE)	LC-MS/MS	BP-3, BP-1, BP-8, BP-2, 4-OH-BP	~200	41–61	70–116	[79]
	Sewage sludge					0.41–0.67	38.3	
DLLME	Lake water Seawater	Magnetic in situ-formed IL	UHPLC-DAD	BP-1, BP-2, BP-3	5	12.3–20.0	68.0–92.5	[83]
LLE	Tap water Stream water Seawater	DEHPA + Fe_3_O_4_	HPLC-UV	BP-1, 4-OH-BP, BP-3	~7	700–800	80–103	[82]
DLLME	Tap water River water Domestic wastewater Factory wastewater	α−terpineol	HPLC-DAD	4OH-BP BP-1 BP BP-4	~20	120–530	75–108.4	[84]

**Table 5:** Sample pretreatment methods not falling into the pretreatment modes of Table 2, Table 3 and Table 4 for BP detection.

Method	Sample	Sorbent	Analytical technique	BPs	Time for Treatment (min)	LOD (ng/L)	Recoveries %	Reference
BAmΕ	Aqueous samples	15 mm—cork-powder-coated polypropylene hollow fibers	HPLC-DAD	BP	120 (15 h for synthesis)	500	100%	[88]
		7.5 mm (half bar)—cork-powder-coated polypropylene hollow fibers				200	123%	
SBSE	Soil	COF-V polypropylene hollow fibers	HPLC-UV	BP-1, BP-3, BP-6, Ph-BP	210–240 (100 h for synthesis)	20–30	73.9–111.7	[90]
BAmΕ	Seawater wastewater	P2-polymer-coated stir bar	HPLC–DAD	BP, BP-3, BP-1, 4-OH-BP	~260	300–500	76.6–103.5	[89]
FPSE	Lake water River water Seawater	Sol–gel-coated sorbent	FPSE-GC/MS	BP-3	20	4.5	94	[91]
QuEChERS	Fish samples (<5% lipids)	MgSO_4_ PSA + Methanol	UHPLC–MS/MS	BP, BP-1, BP-2 BP-3, BP-8, 4-OHBP	~100	0.001–0.122	70–166	[14]
	Fish samples (>5% lipids)						74–182	
Zn-Tb CP	-	CP	Fluorescence	BP	~30 (~96 h for synthesis)	-		[85]
Solid–liquid extraction and SPE	Soil	ethyl acetate–methanol (90%–10%), C18 + anhydrous sodium sulphate	GC-MS	BP-1, BP-3, BP-6, BP-8, 4-OH-BP	~105	0.07–0.10	Soil 89.8–104.4	[86]
	Sediment					0.14–0.28	Sediment 88.4–105.3	
Ultrasound-assisted Solid–liquid extraction	Soils Wastewater treatment plant compost	methanol	UHPLC-MS	BP-1, BP-2, BP-3, BP-6, BP-8	~60	0.05–0.40	83–107	[87]
						0.06–0.30		
